# Glycemic variability and mortality in patients with aortic diseases: A multicenter retrospective cohort study

**DOI:** 10.1371/journal.pone.0325006

**Published:** 2025-06-25

**Authors:** Shanshan Tang, Zhiqiang Zhang, Yu Cheng, Lizhuang Zhang, Qiyao Wang, Cuihua Wang

**Affiliations:** 1 Department of Cardiology, Tianjin Medical University General Hospital, Tianjin Medical University, Tianjin, China; 2 Cardiac Rehabilitation Group, The First Hospital of Hebei Medical University, Shijiazhuang, Hebei, China; Al Nasiriyah Teaching Hospital, IRAQ

## Abstract

**Background:**

The influence of glycemic variability (GV), defined as blood glucose fluctuations, on short-term mortality in patients with aortic diseases, such as aneurysms and dissections, remains understudied. This study evaluates the association between GV and mortality, explores non-linear patterns, and determines a GV threshold predictive of mortality risk.

**Methods:**

A retrospective analysis of 2,441 patients with aortic aneurysm or dissection from the MIMIC IV and eICU-CRD databases was performed. Key variables, including demographics, clinical characteristics, comorbidities, laboratory findings, and treatments, were assessed. Logistic and Cox regression models, smooth curve fitting, and subgroup analyses examined associations between GV and ICU and 30-day mortality.

**Results:**

ICU mortality occurred in 165 patients (6.8%), and 30-day mortality in 235 patients (9.6%). Increased GV was linearly associated with ICU mortality risk (P for non-linearity = 0.666). For 30-day mortality, a U-shaped relationship was observed, with minimal risk at a GV index of 0.2047 (P for non-linearity = 0.041). Adjusted models confirmed these findings (ICU mortality: OR = 1.15, 95% CI = 1.03–1.30, 30-day mortality: HR = 1.10, 95% CI = 1.03–1.18).

**Conclusions:**

GV is significantly associated with short-term mortality in aortic disease patients, with a U-shaped pattern for 30-day mortality and an optimal threshold of 0.2047. These findings underscore the importance of tailored glucose management strategies and call for further research into underlying mechanisms.

## Introduction

Cardiovascular diseases are a major cause of morbidity and mortality worldwide [1]. Aortic aneurysm (AA) is a serious condition characterized by an aortic dilation exceeding 50% of its normal diameter [2]. In many cases, AA remains asymptomatic and undiagnosed in its early stages. However, as the aneurysm enlarges, the risk of life-threatening complications—such as aortic dissection or rupture—increases, with mortality rates ranging from 48% to 56%, even with timely medical intervention [3,4]. Managing these conditions in intensive care units (ICUs) is particularly challenging due to the need for continuous monitoring and intervention to maintain hemodynamic stability and mitigate metabolic and cardiovascular stress.

Patients with aortic aneurysms and dissections in the ICU are at heightened risk due to cardiovascular instability and increased metabolic demands. Many also have comorbidities such as hypertension, diabetes, and metabolic disorders, further complicating their management. These patients frequently experience significant glycemic fluctuations, making glycemic control a critical prognostic factor. Recent studies have highlighted the impact of metabolic parameters, particularly glycemic variability (GV), on cardiovascular disease outcomes [5,6]. GV, defined as fluctuations in blood glucose levels over time, may exert a greater influence on the progression and prognosis of aortic disease. Increased GV has been associated with higher mortality, impaired wound healing, and adverse cardiovascular outcomes, particularly in critically ill patients. Given the intricate relationship between metabolic and cardiovascular factors in aortic disease, investigating the role of GV in short-term mortality and overall prognosis is essential.

This study aims to address a critical research gap by examining the association between GV and mortality in patients with aortic diseases (AD). We hypothesize that increased GV significantly elevates mortality risk in this patient population. Using data from a large, multicenter cohort, we will identify key GV thresholds and nonlinear patterns that influence mortality risk. Understanding these relationships could inform time-sensitive clinical decisions and early intervention strategies, reduce early mortality, and improve clinical outcomes in critically ill patients with aortic pathologies.

## Materials and methods

### Study design and setting

To achieve this, we conducted a detailed retrospective analysis of data from patients diagnosed with aortic aneurysm or dissection using the Medical Information Mart for Intensive Care IV (MIMIC-IV version 2.2) and the eICU Collaborative Research Database (eICU-CRD). Records from MIMIC-IV include over 190,000 patients treated at the Beth Israel Deaconess Medical Center from 2008 to 2019 [1], while eICU-CRD contains data on more than 200,000 ICU patients from over 200 medical centers recorded between 2014 and 2015 [2].

Researchers gained access to these de-identified databases upon successful completion of the Collaborative Institutional Training Initiative (CITI) Program, exemplified by certification numbers 52219361 for Tang and 60071489 for Zhang. The ethical approval for the use of MIMIC-IV data was granted by the Institutional Review Boards of BIDMC and MIT. The de-identified nature of the data negates the necessity for informed consent and ethical approval. After obtaining permission, we downloaded the database locally and used Structured Query Language (SQL) with PostgreSQL (version 13.0) and Navicat software (version 16.0) to identify the cohort and extract the relevant clinical information. Specifically, for clinical parameters with multiple outcomes during a patient’s hospitalization, only the initial outcome was included. Moreover, each variable extraction was double-checked by two individuals to ensure data accuracy and reliability.

Given that this was a retrospective study and all patients were extracted from a public database, informed consent was waived. We adhered to the Strengthening the Reporting of Observational Studies in Epidemiology (STROBE) guidelines [3].

### Patients

**Fig 1:**
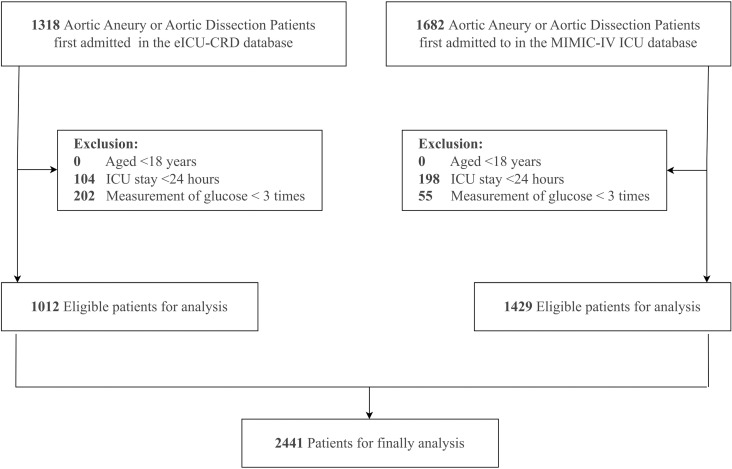
The flowchart of patients’ selection. **Abbreviations:** eICU-CRD, the eICU collaborative research database; MIMIC, medical information mart for intensive care.

Data were extracted using a SQL server for patients fitting the study’s inclusion criteria: adults diagnosed with aortic aneurysm or dissection according to ICD-9 codes 441X or ICD-10 codes I71X (S1 Table), admitted to the ICU. Exclusion criteria included: (1) patients under 18 years of age; (2) those with ICU stays shorter than 24 hours, as this minimum duration was essential to obtain sufficient glucose measurements for reliable glycemic variability assessment; and (3) patients with incomplete triglyceride or glucose data. From an initial population of 3,000 patients (1,318 from eICU-CRD and 1,682 from MIMIC-IV), 302 patients were excluded due to insufficient length of stay or age criteria. Among the remaining patients, those with incomplete glucose measurements were excluded (202 from eICU-CRD and 55 from MIMIC-IV), resulting in a final study cohort of 2,441 patients (1,012 from eICU-CRD and 1,429 from MIMIC-IV) for the analysis (Fig 1).

### Covariates

We included the following variables of enrolled participants in the database based on published literature and clinical experience: demographic characteristics: age, sex, ethnicity. 2) Physical examination: temperature, heart rate, systolic blood pressure (SBP), diastolic blood pressure (DBP). 3) Co-morbidities: hypertension, diabetes, myocardial infarction, congestive heart failure, renal failure. 4) Laboratory tests measured the first time within 24 h of ICU admission: glucose, hemoglobin, WBC count, platelet count, creatinine, and BUN. 5) Medications: statins, angiotensin converting enzyme inhibitor/angiotension receptor blockers (ACEI/ARB), beta-blockers, antiplatelet drugs, vasopressors (including dobutamine, dopamine, phenylephrine, norepinephrine, epinephrine). 6) mechanical ventilation usage. Another factor to consider is the first 24-hour Sequential Organ Failure Assessment (SOFA) score, which was designed to objectively assess the organ function from six aspects: respiration, coagulation, liver, cardiovascular, central nervous system, and renal. Each item was scored 0–4 points, and the higher the score, the more severe the organ dysfunction was reflected [4]. Additionally, we used the Systemic Inflammatory Response Syndrome (SIRS) score, a key clinical tool for evaluating systemic inflammation. A SIRS score of 4 typically signifies a severe inflammatory response, often linked to infections such as sepsis [5].

We computed the mean blood glucose (MBG) of each subject using all available glucose measurements from the first day of ICU admission. To assess glycemic variability, we calculated the coefficient of variation (CV) for glucose concentrations, which is defined as the ratio of the standard deviation (SD) to the MBG. In our cohort, GV values ranged from 0.009 to 1.926, as shown in S1 Fig. To improve the interpretability of this metric, GV was scaled by a factor of 10 in all subsequent analyses. This adjustment means that a one-unit increase in the scaled GV corresponds to a 0.1 increase in the original GV measure (approximately a 10% increase in glycemic variability). The GV values were then stratified into four quartiles for further analysis, with the first quartile serving as the reference group for comparative assessments.

### Outcome

The primary outcome of this study was short-term mortality, encompassing both ICU mortality and mortality within 30 days of ICU admission.

### Statistical analysis

To evaluate the normality of the variable distributions, we utilized histogram distribution analyses, Q-Q plots, and the Kolmogorov-Smirnov test. Continuous variables adhering to a normal distribution were detailed as means ± standard deviation (SD), while those displaying skewness were reported using medians and interquartile ranges (IQR). We presented categorical variables as frequencies and percentages. For group comparisons of continuous variables, we applied either the independent samples Student’s t-test or the Mann-Whitney U test, depending on whether distributions were normal. Categorical data comparisons were performed using either the chi-square test or Fisher’s exact test, as suitable.

Our analysis involved logistic regression to investigate the link between glycemic variability and ICU mortality, and Cox proportional hazards regression to assess its effect on 30-day mortality. We verified hazard proportionality by reviewing “log-log” plots and by integrating survival time interactions. We treated cases lost to follow-up as censored at their last recorded status. Kaplan-Meier survival curves, categorized by levels of glycemic variability, were evaluated using the log-rank test, with glycemic variability divided into quartiles.

The selection of confounding variables was based on clinical relevance, insights from existing literature, importance in univariate analyses, relationships with desired outcomes, or an alteration in the effect estimate of more than 10%. We developed five models: Model 1 without adjustments; Model 2 including demographic adjustments (age, gender, ethnicity); Model 3 with additional vital sign adjustments (temperature, heart rate, systolic blood pressure, diastolic blood pressure) on top of Model 2; Model 4 incorporating comorbidity (hypertension, diabetes, myocardial infarction, congestive heart failure, chronic pulmonary disease, renal failure) and laboratory tests (glucose, hemoglobin, WBC count, platelet count, creatinine, BUN) adjustments in addition to Model 3; and Model 5, the primary model, further adjusted for medication covariates (statins, anti-platelet drugs, ACEI/ARB, beta-blockers, vasopressors) and mechanical ventilation usage on top of Model 4.

To explore potential nonlinear dose-response relationships between glycemic variability and mortality, we employed a restricted cubic spline model. For this, in Model 5, glycemic variability was treated as a continuous variable and set at the 5th, 35th, 65th, and 95th percentiles, as recommended by Harrell [6]. Nonlinearity was examined through a likelihood ratio test comparing a model with a linear term only to another containing both linear and cubic spline terms. When nonlinearity was evident, a two-piecewise regression model was used to pinpoint the threshold effect of glycemic variability on mortality. Subgroup analyses were executed based on designated variables.

Sensitivity analyses were conducted, including exclusions of patients with missing data, those with a SIRS score of 4, and patients with diabetes, to assess the robustness of our results and examine how various inferential models may impact the conclusions, particularly with respect to effect estimates and statistical significance. To account for the potential confounding effect of overall comorbidity burden, we additionally incorporated the Charlson Comorbidity Index (CCI) into our sensitivity analysis. The CCI, a well-established scoring system that quantifies comorbidity burden using weighted scores for specific conditions, has been widely utilized in clinical outcome studies [7]. We reported and compared results, including effect sizes and p-values, across all models to evaluate consistency and robustness.

Details on missing data, which remained under 20% for some variables, are provided in S2 Table.

We reported outcomes as odds ratios (OR) or hazard ratios (HR) with 95% confidence intervals (CI). All statistical procedures were carried out using R Statistical Software (Version 4.2.2) and the Free Statistics Analysis Platform (Version 1.9), which supports reproducible and interactive computing through an intuitive interface powered by R’s statistical engine and Python’s GUI. A two-sided p-value of less than 0.05 was deemed statistically significant for all tests.

## Results

Our study included 2,441 patients who were diagnosed with aortic aneurysms or dissections, with a mean age of 69.2 ± 13.5 years. Of these patients, 70.5% were identified as white and 65.5% as male. The observed ICU mortality rate was 6.8%, and the 30-day mortality rate was 9.6%. Table 1 presents baseline characteristics by GV quartiles. The analysis showed few characteristics remained consistent across groups (age, platelet count, ACEI/ARB usage, all P > 0.05), while most exhibited clear trends with increasing GV. From lowest to highest GV quartiles, disease severity scores significantly increased (SOFA from 4.0 to 6.0, SIRS from 1.9 to 2.5, both P < 0.001), vital signs deteriorated (increased heart rate, decreased blood pressure, P < 0.001), comorbidity proportions rose (hypertension from 31.1% to 53%, heart failure from 12.3% to 21.8%, diabetes from 4.9% to 18.8%, P < 0.05), and treatment requirements increased (vasopressors from 25.2% to 52.7%, mechanical ventilation from 41.6% to 73.6%, P < 0.001). These differences suggest higher GV associates with more complex disease presentations and greater therapeutic needs.

**Table 1:** Baseline characteristics of participants by GV quartiles.

Variables	Total (n = 2441)	Q1 (n = 610)	Q2 (n = 610)	Q3 (n = 610)	Q4 (n = 611)	*P* value
**Database, n (%)**						< 0.001
MIMIC IV	1429 (58.5)	244 (40)	364 (59.7)	417 (68.4)	404 (66.1)	
eICU-CRD	1012 (41.5)	366 (60)	246 (40.3)	193 (31.6)	207 (33.9)	
**Types of aortic aneurysm, n (%)**						0.006
Without rupture	1656 (67.8)	396 (64.9)	426 (69.8)	441 (72.3)	393 (64.3)	
With rupture	785 (32.2)	214 (35.1)	184 (30.2)	169 (27.7)	218 (35.7)	
**Demographics**						
Age, (year)	69.2 ± 13.5	68.8 ± 14.6	69.1 ± 13.3	68.7 ± 13.2	70.3 ± 13.0	0.125
Gender male, n (%)	1600 (65.5)	419 (68.7)	426 (69.8)	393 (64.4)	362 (59.2)	< 0.001
Ethnicity, n (%)						0.005
White	1720 (70.5)	404 (66.2)	435 (71.3)	459 (75.2)	422 (69.1)	
Other	721 (29.5)	206 (33.8)	175 (28.7)	151 (24.8)	189 (30.9)	
**Scores**						
SOFA	5.0 (3.0, 8.0)	4.0 (2.0, 6.0)	5.0 (3.0, 7.0)	6.0 (3.0, 8.0)	6.0 (4.0, 9.0)	< 0.001
SIRS	2.3 ± 1.0	1.9 ± 1.0	2.3 ± 1.0	2.4 ± 1.0	2.5 ± 1.0	< 0.001
CCI	5.3 ± 2.5	4.8 ± 2.4	5.2 ± 2.5	5.3 ± 2.4	5.8 ± 2.7	< 0.001
**Vital signs**						
Temperature, (°C)	36.4 ± 0.9	36.5 ± 0.8	36.4 ± 0.8	36.3 ± 1.0	36.3 ± 1.1	< 0.001
Heart rate, (min^ − 1^)	81.2 ± 16.8	78.9 ± 16.8	80.9 ± 16.2	81.1 ± 16.2	83.7 ± 17.8	< 0.001
Systolic BP, (mmHg)	122.3 ± 25.1	127.3 ± 24.6	121.7 ± 25.5	119.2 ± 23.3	121.1 ± 26.4	< 0.001
Diastolic BP, (mmHg)	66.4 ± 16.3	69.8 ± 16.8	66.6 ± 15.8	64.6 ± 15.7	64.5 ± 16.2	< 0.001
**Co-morbidities, n (%)**						
Hypertension	1137 (46.6)	190 (31.1)	284 (46.6)	339 (55.6)	324 (53)	< 0.001
Myocardial infarction	330 (13.5)	66 (10.8)	77 (12.6)	77 (12.6)	110 (18)	0.002
Congestive heart failure	417 (17.1)	75 (12.3)	94 (15.4)	115 (18.9)	133 (21.8)	< 0.001
Diabetes	266 (10.9)	30 (4.9)	52 (8.5)	69 (11.3)	115 (18.8)	< 0.001
Chronic pulmonary disease	587 (24.0)	129 (21.1)	145 (23.8)	156 (25.6)	157 (25.7)	0.21
Renal failure	322 (13.2)	63 (10.3)	76 (12.5)	82 (13.4)	101 (16.5)	0.014
**Laboratory tests**						
Glucose, (mg/dl)^*^	139.3 ± 50.3	118.4 ± 24.9	131.3 ± 30.7	140.8 ± 38.4	166.5 ± 76.3	< 0.001
Mean blood glucose, (mg/dl)^#^	125.6 ± 23.4	114.8 ± 20.1	122.2 ± 18.1	127.1 ± 19.6	138.3 ± 28.1	< 0.001
Hemoglobin, (g/dl)	10.4 (9.1, 11.9)	10.9 (9.4, 12.2)	10.3 (9.0, 12.0)	10.2 (9.0, 11.5)	10.1 (8.9, 11.7)	< 0.001
WBC, (K/μL)	11.4 ± 5.1	10.3 ± 4.4	11.4 ± 4.8	11.8 ± 5.5	12.1 ± 5.5	< 0.001
Platelet, (K/μl)	168.0 (124.0, 226.0)	167.0 (128.0, 224.0)	167.0 (126.2, 223.8)	169.5 (124.0, 222.5)	171.0 (117.5, 230.5)	0.744
Creatinine, (mg/dl)	1.0 (0.8, 1.3)	1.0 (0.8, 1.3)	1.0 (0.8, 1.3)	1.0 (0.7, 1.3)	1.1 (0.8, 1.5)	0.012
BUN, (mg/dl)	18.0 (13.0, 25.0)	17.0 (13.0, 24.0)	17.5 (13.2, 24.0)	18.0 (14.0, 24.0)	19.0 (14.0, 27.0)	0.003
**Medications, n (%)**						
Statin	896 (36.7)	196 (32.1)	238 (39)	251 (41.1)	211 (34.5)	0.004
Anti-Platelet drug	1316 (53.9)	274 (44.9)	338 (55.4)	381 (62.5)	323 (52.9)	< 0.001
ACEI/ARB	450 (18.4)	126 (20.7)	109 (17.9)	112 (18.4)	103 (16.9)	0.37
Beta-blockers	1443 (59.1)	310 (50.8)	374 (61.3)	409 (67)	350 (57.3)	< 0.001
Vasopressor	1128 (46.2)	154 (25.2)	299 (49)	353 (57.9)	322 (52.7)	< 0.001
Mechanical ventilation	1569 (64.3)	254 (41.6)	403 (66.1)	462 (75.7)	450 (73.6)	< 0.001
**Outcomes**						
Length of hospital stay, (days)	7.3 (4.9, 12.0)	5.9 (4.0, 9.1)	7.1 (5.0, 11.9)	8.1 (5.5, 13.6)	8.7 (5.2, 14.6)	< 0.001
ICU mortality, n (%)	165 (6.8)	17 (2.8)	38 (6.2)	30 (4.9)	80 (13.1)	< 0.001
Hospital mortality, n (%)	221 (9.1)	31 (5.1)	48 (7.9)	42 (6.9)	100 (16.4)	< 0.001
30-day mortality, n (%)	235 (9.6)	40 (6.6)	46 (7.5)	44 (7.2)	105 (17.2)	< 0.001

**Abbreviations:** MIMIC, medical information mart for intensive care; eICU-CRD, the eICU collaborative research database; SOFA, sequential organ failure assessment; SIRS, systemic inflammatory response syndrome; GCS, glasgow coma scale; BP, blood pressure; GV, glycemic variability; WBC, white blood cell; BUN, blood urea nitrogen; ACEI/ARB, angiotensin converting enzyme inhibitors/angiotension receptor blockers.Note:

* : refers to the first blood glucose measurement upon ICU admission;

# : refers to the average blood glucose level during the ICU stay.

### ICU mortality

Univariate and multivariable logistic regression analyses were conducted to evaluate risk factors for ICU mortality among patients with aortic diseases, as presented in S3 Table. Univariate analysis identified significant associations with temperature (OR 0.76, P < 0.001), heart rate (OR 1.02, P < 0.001), Sequential Organ Failure Assessment (SOFA) score (OR 1.30, P < 0.001), use of anti-platelet drugs (OR 0.46, P < 0.001), and beta-blockers (OR 0.38, P < 0.001), which persisted after adjustment for various confounders.

Adjusted smoothed plots demonstrated a linear relationship between glycemic variability and ICU mortality (Fig 3, P for non-linearity = 0.666, excluding the highest and lowest 0.5% of data points for each glycemic variability measure). An increase in glycemic variability corresponded with a higher risk of ICU mortality.

**Fig 2:**
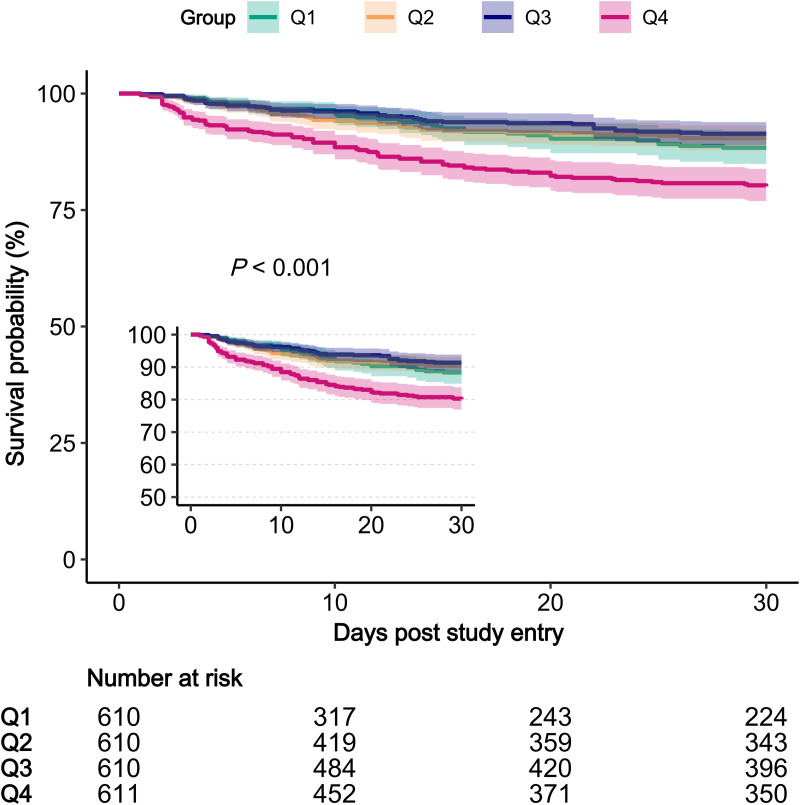
Kaplan-Meier survival curves for 30-day mortality.

Multivariable logistic regression analysis, adjusting for confounders specified in Table 1, treated glycemic variability as a continuous variable. Each 0.1 unit increase in GV was inversely associated with mortality risk (OR = 1.32, 95% CI = 1.20–1.45, *P* < 0.001). Patients in the highest quartile of glycemic variability faced significantly elevated odds of ICU mortality compared to those in the lowest quartile (OR: 2.8, 95% CI: 1.53–5.16), detailed in Table 2.

**Table 2:** Associations between GV and ICU mortality in the multiple regression model.

Models	GV*10		GV quartiles			
	(n = 2441)		Q1 (n = 610)	Q2 (n = 610)	Q3 (n = 610)	Q4 (n = 611)
	OR (95% CI)	*P* value	OR (95% CI)	OR (95% CI)	OR (95% CI)	OR (95% CI)
Model 1	1.32 (1.20, 1.45)	<0.001	1.00 (Ref)	2.32 (1.29, 4.15)	1.80 (0.98, 3.31)	5.26 (3.07, 8.99)
Model 2	1.31 (1.19, 1.43)	<0.001	1.00 (Ref)	2.36 (1.32, 4.24)	1.86 (1.01, 3.42)	5.14 (3.00, 8.81)
Model 3	1.16 (1.04, 1.29)	0.006	1.00 (Ref)	2.00 (1.08, 3.68)	1.40 (0.73, 2.67)	3.54 (2.00, 6.26)
Model 4	1.18 (1.05, 1.32)	0.004	1.00 (Ref)	2.03 (1.08, 3.82)	1.24 (0.64, 2.39)	3.10 (1.70, 5.65)
Model 5	1.15 (1.03, 1.30)	0.018	1.00 (Ref)	2.04 (1.08, 3.85)	1.29 (0.66, 2.52)	2.81 (1.53, 5.16)

**Abbreviations**: OR, odds ratio; CI, confidence interval. **Note:** Model 1 adjusted for: none; Model 2 adjusted for: covariates included in demographics (age, gender, ethnicity); Model 3 adjusted for: model 2 + covariates included in vital signs (temperature, heart rate, systolic blood pressure, diastolic blood pressure); Model 4 adjusted for: model 3 + covariates included in co-morbidities(hypertension, diabetes, myocardial infarction, congestive heart failure, chronic pulmonary disease, renal failure)+ laboratory tests (glucose, hemoglobin, WBC count, platelet count, creatinine, BUN); Model 5 adjusted for: model 4 + covariates included in medications (statin use, anti-platelet drugs, ACEI/ARB, beta-blockers, vasopressor use, and mechanical ventilation).

### 30-day mortality

The log-rank test revealed significant differences between the survival curves, indicating that patients with the highest levels of glycemic variability (GV) experienced notably lower 30-day survival rates compared to those with lower levels (p < 0.001) (Fig 2). This finding underscores a worse prognosis for patients with aortic diseases who have higher GV.

Univariate and multivariable Cox regression analyses were utilized to identify risk factors for 30-day mortality among patients with aortic diseases, with results shown in S4 Table. Key associations were noted with factors such as gender (HR 0.73, P = 0.017), heart rate (HR 1.02, P < 0.001), the Sequential Organ Failure Assessment (SOFA) score (HR 1.19, P < 0.001), use of anti-platelet drugs (HR 0.39, P < 0.001), and beta-blockers (HR 0.30, P < 0.001), and these remained significant even after adjustments for other confounders.

A smoothing function analysis explored the dose-response relationship between GV levels and the occurrence of 30-day mortality. This analysis, conducted using restricted cubic splines, depicted a U-shaped relationship, with marked nonlinearity (P < 0.041, Fig 4). The point of lowest risk was pinpointed at a GV value of 0.2047, determined by a two-piecewise linear regression model. Below this inflection point, an increase in GV was associated with a decrease in mortality risk (HR: 0.004, 95% CI: 0, 3.08, P = 0.103); however, above it, the relationship inverted (HR: 2.19, 95% CI: 1.01–4.74, P = 0.047), even after accounting for all relevant covariates. These outcomes imply that both extremely low and high GV levels correlate with an increased risk of 30-day mortality (see Table 4).

**Table 3:** Associations between GV and 30-day mortality in the multiple regression model.

Models	GV*10		GV quartiles			
	(n = 2441)		Q1 (n = 610)	Q2 (n = 610)	Q3 (n = 610)	Q4 (n = 611)
	HR (95% CI)	*P* value	HR (95% CI)	HR (95% CI)	HR (95% CI)	HR (95% CI)
Model 1	1.18 (1.12, 1.25)	<0.001	1.00 (Ref)	0.93 (0.61, 1.41)	0.81 (0.52, 1.24)	2.06 (1.43, 2.96)
Model 2	1.17 (1.11, 1.24)	<0.001	1.00 (Ref)	0.95 (0.62, 1.45)	0.84 (0.54, 1.29)	2.03 (1.41, 2.92)
Model 3	1.16 (1.09, 1.24)	<0.001	1.00 (Ref)	0.89 (0.58, 1.36)	0.75 (0.49, 1.16)	1.76 (1.21, 2.55)
Model 4	1.15 (1.09, 1.23)	<0.001	1.00 (Ref)	1.00 (0.65 ~ 1.55)	0.83 (0.53, 1.30)	2.00 (1.35, 2.96)
Model 5	1.10 (1.03, 1.18)	<0.001	1.00 (Ref)	1.01 (0.65, 1.56)	0.85 (0.54, 1.33)	1.74 (1.17, 2.59)

**Abbreviations**: HR, hazard ratio; CI, confidence interval. **Note:** Model 1 adjusted for: none; Model 2 adjusted for: covariates included in demographics (age, gender, ethnicity); Model 3 adjusted for: model 2 + covariates included in vital signs (temperature, heart rate, systolic blood pressure, diastolic blood pressure); Model 4 adjusted for: model 3 + covariates included in co-morbidities(hypertension, diabetes, myocardial infarction, congestive heart failure, chronic pulmonary disease, renal failure)+ laboratory tests (glucose, hemoglobin, WBC count, platelet count, creatinine, BUN); Model 5 adjusted for: model 4 + covariates included in medications (statin use, anti-platelet drugs, ACEI/ARB, beta-blockers, vasopressor use, and mechanical ventilation).

**Table 4:** The non-linearity relationship between GV and 30-day mortality.

Threshold of GV	HR	95% CI	*P*-value
<0.2047	0.004	(0, 3.08)	0.103
≥0.2047	15.5	(2.9, 82.8)	0.001
Non-linear test			0.135

**Abbreviations:** HR, hazard ratio; CI, confidence interval. **Note:** Adjusted for the following covariates: demographics (age, gender, ethnicity); vital signs (temperature, heart rate, systolic blood pressure, diastolic blood pressure); comorbidities (hypertension, diabetes, myocardial infarction, congestive heart failure, chronic pulmonary disease, renal failure); laboratory tests (glucose, hemoglobin, WBC count, platelet count, creatinine, BUN); and medications (statin use, anti-platelet drugs, ACEI/ARB, beta-blockers, vasopressor use, and mechanical ventilation).

**Fig 3:**
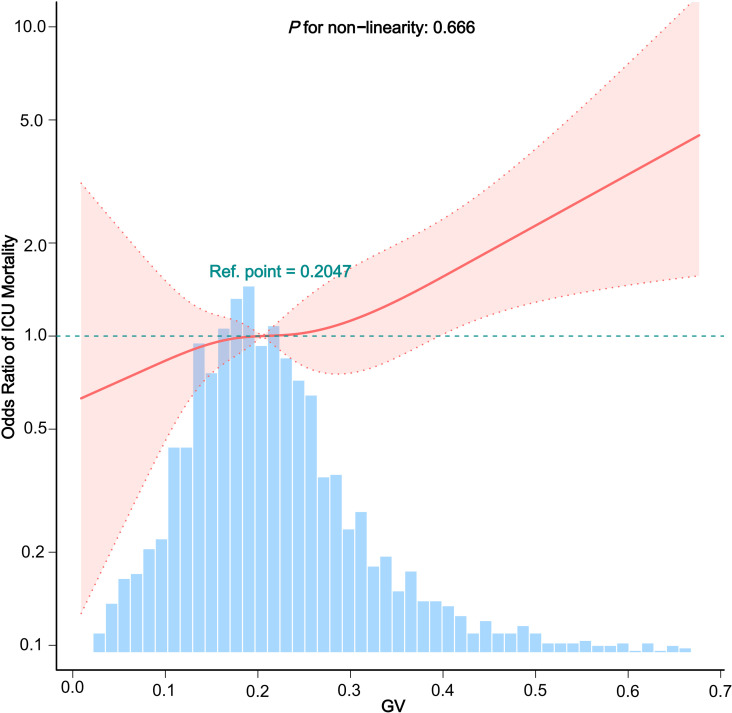
Dose-response relationship between the GV and ICU mortality. **Note:** Adjusted for the following covariates: demographics (age, gender, ethnicity); vital signs (temperature, heart rate, systolic blood pressure, diastolic blood pressure); comorbidities (hypertension, diabetes, myocardial infarction, congestive heart failure, chronic pulmonary disease, renal failure); laboratory tests (glucose, hemoglobin, WBC count, platelet count, creatinine, BUN); and medications (statin use, anti-platelet drugs, ACEI/ARB, beta-blockers, vasopressor use, and mechanical ventilation).

**Fig 4:**
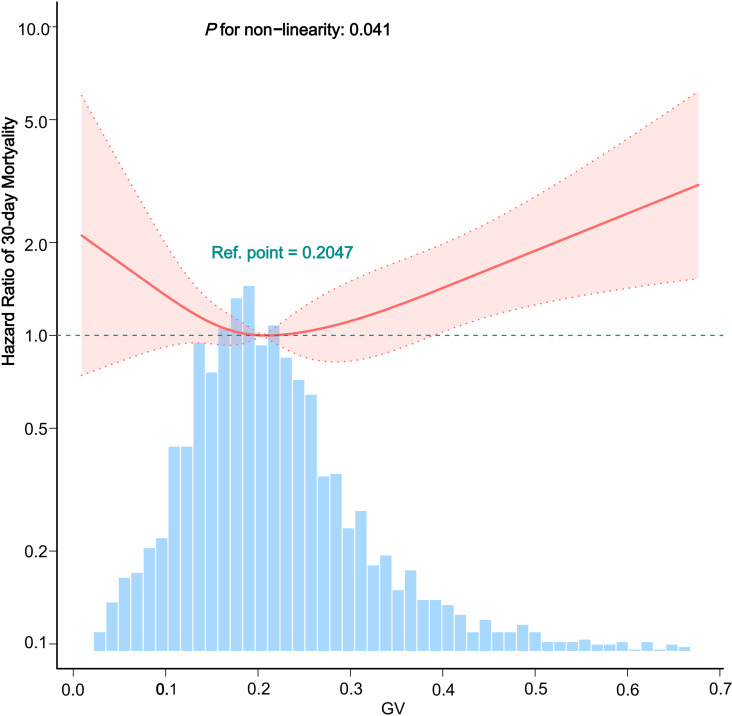
Dose-response relationship between the GV and 30-day mortality. **Note:** Adjusted for the following covariates: demographics (age, gender, ethnicity); vital signs (temperature, heart rate, systolic blood pressure, diastolic blood pressure); comorbidities (hypertension, diabetes, myocardial infarction, congestive heart failure, chronic pulmonary disease, renal failure); laboratory tests (glucose, hemoglobin, WBC count, platelet count, creatinine, BUN); and medications (statin use, anti-platelet drugs, ACEI/ARB, beta-blockers, vasopressor use, and mechanical ventilation).

In multivariable logistic regression, after adjusting for the confounders outlined in in Table 1, GV treated as a continuous variable, inversely correlated with the risk of mortality (HR = 1.18, 95% CI = 1.12–1.25). Patients in the highest quartile of GV show significantly different ICU mortality odds compared to those in the lowest quartile (HR: 1.74, 95% CI: 1.17–2.59) (Table 3).

S2 Fig demonstrates that, even after excluding patients with missing data, a SIRS score of 4, and diabetes, the observed association between aortic pathologies and mortality remained unchanged. This finding indicates that imputing missing data, along with the presence of diabetes and systemic inflammation, had no substantial impact on our primary conclusions. Furthermore, when adjusting for the Charlson Comorbidity Index (CCI) in the sensitivity analysis, the association persisted, suggesting that the burden of comorbidities, as captured by the CCI, did not significantly alter the relationship between aortic pathologies and mortality.

Finally, to assess potential modifications in the association between glycemic variability and 30-day mortality, a stratified analysis was conducted across multiple subgroups (S3 Fig). Following stratification by factors such as AD type, age, sex, ethnicity, hypertension, diabetes, myocardial infarction, congestive heart failure, chronic pulmonary disease, eGFR, and SIRS, no statistically significant interactions were identified in any subgroup.

## Discussion

To our knowledge, this is the first retrospective cohort study utilizing MIMIC IV and eICU-CRD data to demonstrate a 119% increase in 30-day mortality risk in aortic disease patients associated with glycemic variability (GV). Restricted cubic spline analysis revealed a U-shaped relationship between GV and 30-day mortality risk (P for nonlinearity <0.05), with the lowest risk at a GV of 0.204. This U-shaped association was confirmed in subsequent subgroup analyses. Additionally, a linear relationship was observed between GV and ICU mortality, indicating that increased GV correlates with higher ICU mortality risk. These findings have significant clinical implications.

Previous studies have identified GV as an independent risk factor for mortality in critically ill patients. [8–11]. For example, in patients with acute stroke, Lin et al. found that higher acute GV is a risk factor for early mortality in acute stroke patients [9]. GV is also indicative of metabolic instability and poor glycemic control, often leading to greater oxidative stress, higher inflammatory cytokine levels, and more severe endothelial dysfunction compared to chronic hyperglycemia [12,13]. Although our study demonstrates a significant association between GV and mortality in patients with aortic disease, the exact mechanisms remain to be fully elucidated. It is possible that cardiovascular complications, such as acute coronary events, arrhythmias, or thromboembolic events, contributed to the increased mortality risk. However, due to dataset limitations, we were unable to directly ascertain the specific causes of death. Future studies with more granular clinical data are needed to further investigate this potential association.

Known risk factors for aortic aneurysm (AA) include male gender, Caucasian race, smoking, hypertension, elevated LDL, and hypercholesterolemia [14–17]. Few studies have explored the relationship between GV and mortality in aortic disease patients. We observed a U-shaped association with 30-day mortality even after adjusting for age, gender, ethnicity, comorbidities, and treatment. Future studies are needed to validate these findings and investigate the underlying mechanisms in more detail.

Our study demonstrated a significant association between GV and 30-day mortality in patients with aortic disease. While the two-piecewise linear regression model detected a U-shaped trend, this effect was mainly influenced by extreme values. The primary relationship remains linear, supporting the hypothesis that higher GV is associated with increased mortality risk. In contrast, GV was not significantly linked to ICU mortality, indicating that its impact on short-term and mid-term outcomes may differ. Although the associations between GV and ICU mortality were modest, a linear relationship indicated that increased GV is associated with higher ICU mortality risk. This study is the first to provide comprehensive insights into the association between GV and mortality risk in aortic disease patients, using robust data from the MIMIC IV and eICU-CRD databases.

Glycemic variability is associated with heightened mortality risks in patients suffering from aortic aneurysms or dissections due to several underlying mechanisms: (1) Oxidative stress and inflammation: Variations in blood glucose levels provoke oxidative stress and trigger inflammatory reactions, damaging the vascular walls and hastening the progression of aortic conditions [13]. (2) Vascular smooth muscle cell dysfunction: fluctuations in blood glucose adversely affect the functionality of vascular smooth muscle cells, leading to a weakened aortic wall [13]. (3) Endothelial dysfunction: inconsistent glucose levels compromise endothelial function, hindering the production of vital molecules such as nitric oxide, essential for maintaining vascular health [18,19]. These observations underscore that glycemic variability not only directly impacts the structural integrity of aortic aneurysms and dissections but also intensifies these conditions through the subtle modulation of inflammation and endothelial function. Future studies should explore these mechanisms in detail and assess how managing glycemic fluctuations can enhance patient outcomes and disease management strategies.

Our study shows that GV impacts mortality risk in aortic disease patients. Monitoring GV alongside average glucose levels helps identify high-risk patients. Managing GV may reduce oxidative stress, inflammation, and disease progression. Early detection allows for timely interventions like adjusting insulin therapy or lifestyle changes, improving outcomes. We recommend including GV in treatment guidelines for better long-term management.

However, several key limitations warrant acknowledgment. As an observational analysis, this study cannot establish definitive causal relationships. Despite employing regression models, stratified evaluations, and sensitivity assessments, residual confounding remains possible. Additionally, the MIMIC database structure presents inherent challenges in isolating primary admission diagnoses. Moreover, we were unable to reliably distinguish between pre-admission and post-admission diagnoses, which may have affected our ability to accurately assess the temporal relationship between comorbidities, glycemic variability, and mortality outcomes. Thirdly, the absence of complete HbA1c measurements in the eICU database limited our ability to comprehensively assess its role as a glycemic marker in relation to mortality. Despite our attempts to incorporate HbA1c data from up to 90 days prior to MIMIC-IV ICU admission, 40.27% of the data remained unavailable. This limitation, which has also been reported in studies utilizing the MIMIC-IV database, may affect the accuracy of our findings, particularly in terms of long-term glycemic control and mortality outcomes. Finally, the study’s predominantly U.S.-based population may limit the generalizability of findings and statistical power. Despite these constraints, our results provide valuable insights into the relationship between glycemic variability and mortality in aortic disease patients, highlighting the need for validation in larger, more diverse cohorts.

## Conclusions

This study shows that elevated GV is linked to higher 30-day mortality in aortic disease patients. Both low and high GV levels increase ICU mortality, highlighting GV as a key risk factor. Managing GV could improve outcomes by reducing oxidative stress and inflammation. Further research is needed to explore how targeting GV through therapies or lifestyle changes can enhance survival.

## Supporting information

S1 Fig Distribution and summary statistics of glycemic variability. (PDF)

S2 Fig Sensitivity analysis between GV and 30-day mortality. **Abbreviations:** HR, hazard ratio; CI, confidence interval; SIRS, systemic inflammatory response syndrome. **Note:** Adjusted for the following covariates: demographics (age, gender, ethnicity); vital signs (temperature, heart rate, systolic blood pressure, diastolic blood pressure); comorbidities (hypertension, diabetes, myocardial infarction, congestive heart failure, chronic pulmonary disease, renal failure); laboratory tests (glucose, hemoglobin, WBC count, platelet count, creatinine, BUN); and medications (statin use, anti-platelet drugs, ACEI/ARB, beta-blockers, vasopressor use, and mechanical ventilation).
(PDF)

S3 Fig Subgroup analysis of 30-day mortality. **Abbreviations:** HR, hazard ratio; CI, confidence interval; eGFR, estimated glomerular filtration rate; SIRS, systemic inflammatory response syndrome. **Note:** Adjusted for the following covariates: demographics (age, gender, ethnicity); vital signs (temperature, heart rate, systolic blood pressure, diastolic blood pressure); comorbidities (hypertension, diabetes, myocardial infarction, congestive heart failure, chronic pulmonary disease, renal failure); laboratory tests (glucose, hemoglobin, WBC count, platelet count, creatinine, BUN); and medications (statin use, anti-platelet drugs, ACEI/ARB, beta-blockers, vasopressor use, and mechanical ventilation).
(PDF)

S1 Table ICD code for aortic aneurysm and dissection. (DOCX)

S2 Table Missing rates of study variables. (DOCX)

S3 Table Univariate and multivariable logistic analysis evaluating the association between GV and ICU mortality. (DOCX)

S4 Table Univariate and multivariable Cox analysis evaluating the association between GV and 30-day mortality. (DOCX)
