# Supplementary figures and images for: Glycemic variability and mortality in patients with aortic diseases: A multicenter retrospective cohort study
Source: PLoS One. 2025 Jun 25;20(6):e0325006. doi: 10.1371/journal.pone.0325006 (PMC12193046; doi:10.1371/journal.pone.0325006)

Sorted by GV values

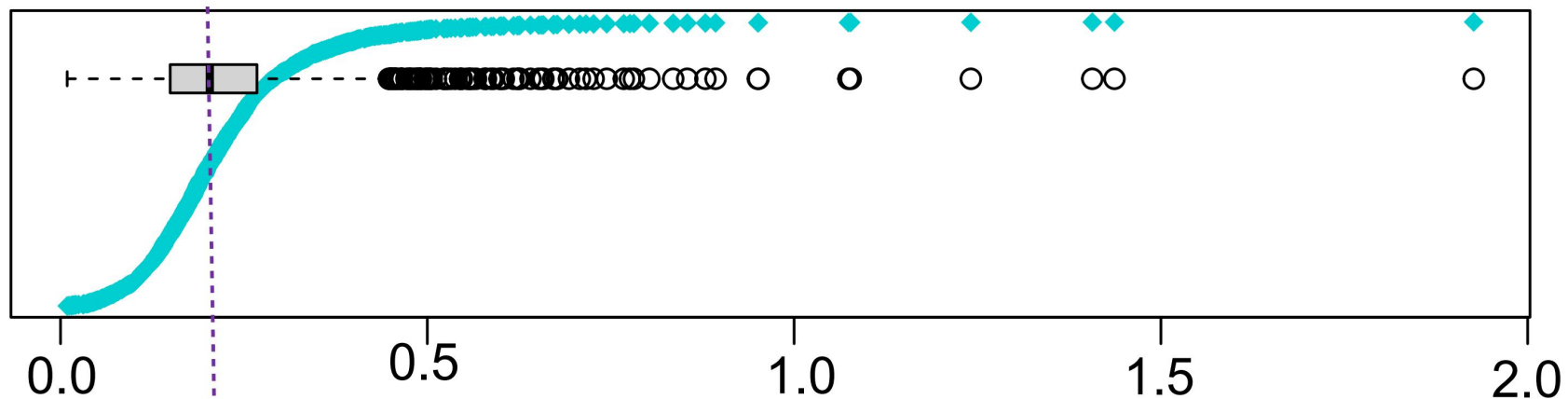

| obs. | mean  | median | s.d.  | min.  | max.  |
|------|-------|--------|-------|-------|-------|
| 2441 | 0.225 | 0.203  | 0.127 | 0.009 | 1.926 |

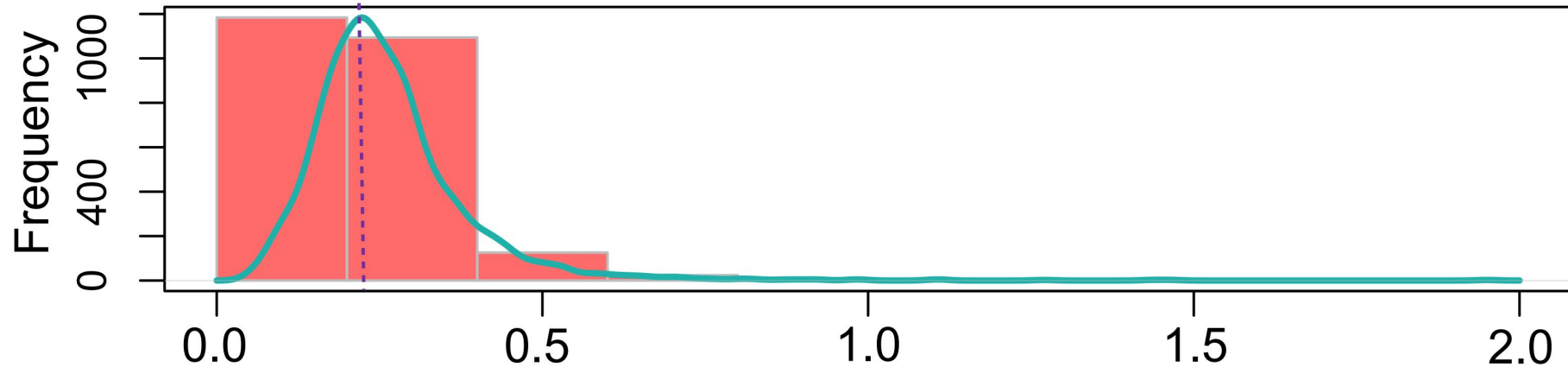

Supplement: S1 Fig — (PDF) [file pone.0325006.s001.pdf]

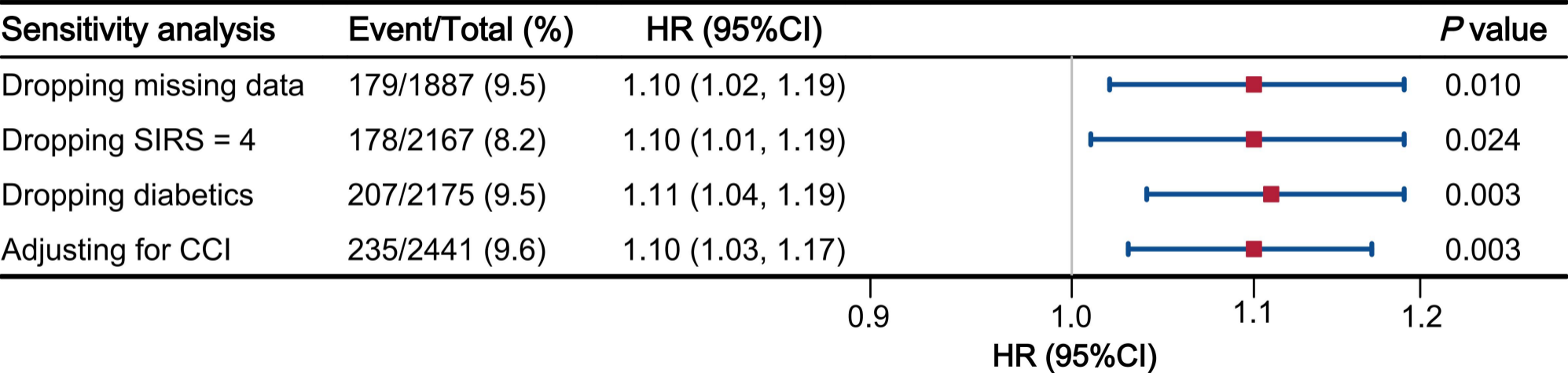

Supplement: S2 Fig — Abbreviations: HR, hazard ratio; CI, confidence interval; SIRS, systemic inflammatory response syndrome. Note: Adjusted for the following covariates: demographics (age, gender, ethnicity); vital signs (temperature, heart rate, systolic blood pressure, diastolic blood pressure); comorbidities (hypertension, diabetes, myocardial infarction, congestive heart failure, chronic pulmonary disease, renal failure); laboratory tests (glucose, hemoglobin, WBC count, platelet count, creatinine, BUN); and medications (statin use, anti-platelet drugs, ACEI/ARB, beta-blockers, vasopressor use, and mechanical ventilation). (PDF) [file pone.0325006.s002.pdf]

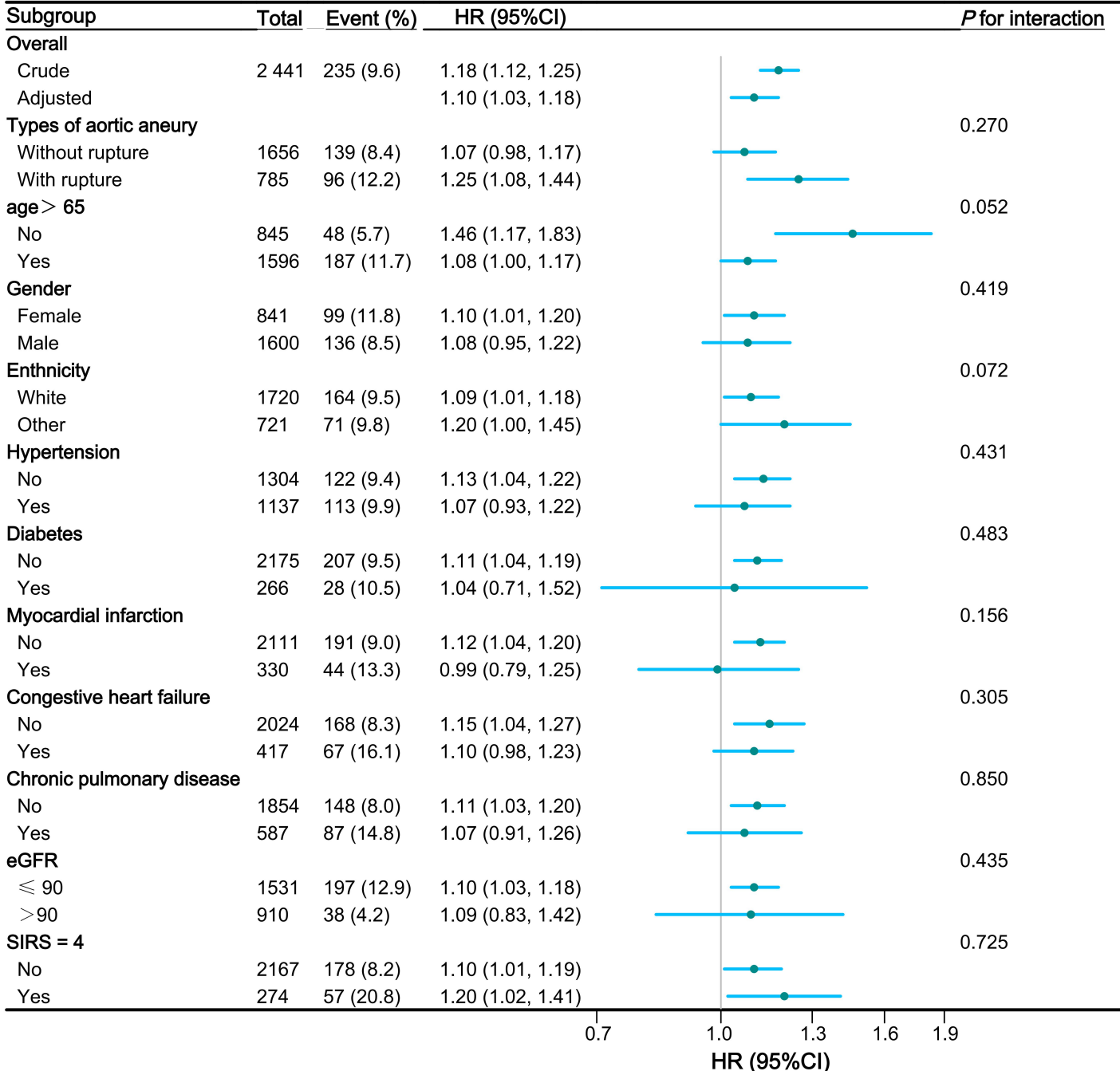

Supplement: S3 Fig — Abbreviations: HR, hazard ratio; CI, confidence interval; eGFR, estimated glomerular filtration rate; SIRS, systemic inflammatory response syndrome. Note: Adjusted for the following covariates: demographics (age, gender, ethnicity); vital signs (temperature, heart rate, systolic blood pressure, diastolic blood pressure); comorbidities (hypertension, diabetes, myocardial infarction, congestive heart failure, chronic pulmonary disease, renal failure); laboratory tests (glucose, hemoglobin, WBC count, platelet count, creatinine, BUN); and medications (statin use, anti-platelet drugs, ACEI/ARB, beta-blockers, vasopressor use, and mechanical ventilation). (PDF) [file pone.0325006.s003.pdf]
