# Supplementary material for: Glycemic variability and mortality in patients with aortic diseases: A multicenter retrospective cohort study
Source: PLoS One. 2025 Jun 25;20(6):e0325006. doi: 10.1371/journal.pone.0325006 (PMC12193046; doi:10.1371/journal.pone.0325006)
Supplement: S1 Table — (DOCX) [file pone.0325006.s004.docx]

**TableS1** ICD code for aortic aneurysm and dissection

| ICD version | ICD code | Long title |
| --- | --- | --- |
| 9 | 44100 | Dissection of aorta, unspecified site |
|  | 44101 | Dissection of aorta, thoracic |
|  | 44102 | Dissection of aorta, abdominal |
|  | 44103 | Dissection of aorta, thoracoabdominal |
|  | 4411 | Thoracic aneurysm, ruptured |
|  | 4412 | Thoracic aneurysm without mention of rupture |
|  | 4413 | Abdominal aneurysm, ruptured |
|  | 4414 | Abdominal aneurysm without mention of rupture |
|  | 4415 | Aortic aneurysm of unspecified site, ruptured |
|  | 4416 | Thoracoabdominal aneurysm, ruptured |
|  | 4417 | Thoracoabdominal aneurysm, without mention of rupture |
|  | 4419 | Aortic aneurysm of unspecified site without mention of rupture |
| 10 | I71 | Aortic aneurysm and dissection |
|  | I710 | Dissection of aorta |
|  | I7100 | Dissection of unspecified site of aorta |
|  | I7101 | Dissection of thoracic aorta |
|  | I7102 | Dissection of abdominal aorta |
|  | I7103 | Dissection of thoracoabdominal aorta |
|  | I711 | Thoracic aortic aneurysm, ruptured |
|  | I712 | Thoracic aortic aneurysm, without rupture |
|  | I713 | Abdominal aortic aneurysm, ruptured |
|  | I714 | Abdominal aortic aneurysm, without rupture |
|  | I715 | Thoracoabdominal aortic aneurysm, ruptured |
|  | I716 | Thoracoabdominal aortic aneurysm, without rupture |
|  | I718 | Aortic aneurysm of unspecified site, ruptured |
|  | I719 | Aortic aneurysm of unspecified site, without rupture |
