# Supplementary material for: Glycemic variability and mortality in patients with aortic diseases: A multicenter retrospective cohort study
Source: PLoS One. 2025 Jun 25;20(6):e0325006. doi: 10.1371/journal.pone.0325006 (PMC12193046; doi:10.1371/journal.pone.0325006)
Supplement: S2 Table — (DOCX) [file pone.0325006.s005.docx]

**Table S2.** Missing rates of study variables

| **Variables** | **Missing, (%)**  ***n*=2441** |
| --- | --- |
| Temperature | 4.42 |
| Heart rate | 3.11 |
| Systolic BP | 5.16 |
| Systolic BP | 5.16 |
| Hemoglobin | 8.19 |
| WBC | 0.33 |
| Platelet | 8.68 |
| Creatinine | 2.95 |
| BUN | 3.28 |

**Abbreviations:** BP, blood pressure; WBC, white blood cell; BUN, blood urea nitrogen.
