# Supplementary material for: Glycemic variability and mortality in patients with aortic diseases: A multicenter retrospective cohort study
Source: PLoS One. 2025 Jun 25;20(6):e0325006. doi: 10.1371/journal.pone.0325006 (PMC12193046; doi:10.1371/journal.pone.0325006)
Supplement: S3 Table — (DOCX) [file pone.0325006.s006.docx]

**Table S3** Univariate and multivariable logistic analysis evaluating the association between GV and ICU mortality.

| **Variable** | **Univariate logistic analysis** | | **Multivariable logistic analysis** | |
| --- | --- | --- | --- | --- |
|  | **OR (95%CI)** | ***P*-value** | **OR (95%CI)** | ***P*-value** |
| **Demographics** | | | | |
| Age | 1.02 (1.01, 1.03) | 0.006 | 1.01 (1.00, 1.03) | 0.080 |
| Gender | 0.73 (0.53, 1.01) | 0.059 | 0.65 (0.44, 0.96) | 0.032 |
| Ethnicity | 1.32 (0.95, 1.84) | 0.103 | 1.23 (0.82, 1.85) | 0.315 |
| **Vital signs** | | | | |
| Temperature | 0.76 (0.66, 0.88) | <0.001 | 0.85 (0.72, 0.99) | 0.041 |
| Heart rate | 1.02 (1.01, 1.03) | <0.001 | 1.01 (1.00, 1.02) | 0.035 |
| Systolic BP | 0.99 (0.99, 1.00) | 0.104 | 1.00 (1.00, 1.01) | 0.339 |
| Systolic BP | 0.99 (0.98, 1.00) | 0.058 | 1.00 (0.98, 1.01) | 0.843 |
| SOFA | 1.30 (1.25, 1.36) | <0.001 | 1.28 (1.21, 1.35) | <0.001 |
| **Co-morbidities** | | | | |
| Hypertension | 1.24 (0.90, 1.70) | 0.189 | 0.64 (0.37, 1.10) | 0.107 |
| Myocardial infarction | 1.46 (0.97, 2.21) | 0.071 | 1.29 (0.78, 2.13) | 0.318 |
| Congestive heart failure | 1.61 (1.11, 2.34) | 0.012 | 0.93 (0.57, 1.53) | 0.786 |
| Diabetes | 1.00 (0.60, 1.66) | 0.996 | 0.84 (0.45, 1.57) | 0.589 |
| Renal failure | 1.79 (1.20, 2.66) | 0.004 | 0.69 (0.38, 1.24) | 0.214 |
| **Medications** | | | | |
| Statin | 0.44 (0.30, 0.65) | <0.001 | 0.66 (0.41, 1.07) | 0.093 |
| Anti-platelet drugs | 0.46 (0.33, 0.64) | <0.001 | 0.57 (0.37, 0.87) | 0.009 |
| ACEI/ARB | 0.30 (0.16, 0.56) | <0.001 | 0.61 (0.31, 1.22) | 0.165 |
| Beta-blockers | 0.38 (0.27, 0.53) | <0.001 | 0.58 (0.39, 0.86) | 0.007 |
| Vasopressor | 1.68 (1.22, 2.31) | 0.002 | 0.93 (0.58, 1.49) | 0.769 |
| Mechanical ventilation | 2.16 (1.47, 3.17) | <0.001 | 1.80 (0.92, 3.52) | 0.086 |
| GV | 15.82 (6.22, 40.29) | <0.001 | 3.21 (0.99, 10.38) | 0.052 |

**Abbreviations:** OR, odds ratio; CI, confidence interval; BP, blood pressure; SOFA, sequential organ failure assessment; ACEI/ARB, angiotensin converting enzyme inhibitors/angiotension receptor blockers; GV, glycemic variability.

**Note:** multivariable logistic analysis adjusted for covariates: demographics (age, gender, ethnicity); vital signs (temperature, heart rate, systolic blood pressure, diastolic blood pressure); comorbidities (hypertension, diabetes, myocardial infarction, congestive heart failure, chronic pulmonary disease, renal failure); laboratory tests (glucose, hemoglobin, WBC count, platelet count, creatinine, BUN); and medications (statin use, anti-platelet drugs, ACEI/ARB, beta-blockers, vasopressor use, and mechanical ventilation).
