# Supplementary material for: Glycemic variability and mortality in patients with aortic diseases: A multicenter retrospective cohort study
Source: PLoS One. 2025 Jun 25;20(6):e0325006. doi: 10.1371/journal.pone.0325006 (PMC12193046; doi:10.1371/journal.pone.0325006)
Supplement: S4 Table — (DOCX) [file pone.0325006.s007.docx]

**Table S4** Univariate and multivariable Cox analysis evaluating the association between GV and 30-day mortality.

| **Variable** | **Univariate Cox analysis** | | **Multivariable Cox analysis** | |
| --- | --- | --- | --- | --- |
|  | **HR (95%CI)** | ***P*-value** | **HR (95%CI)** | ***P*-value** |
| **Demographics** | | | | |
| Age | 1.03 (1.02, 1.04) | <0.001 | 1.03 (1.01, 1.04) | <0.001 |
| Gender | 0.73 (0.56, 0.95) | 0.017 | 0.67 (0.5, 0.89) | 0.006 |
| Ethnicity | 1.07 (0.81, 1.42) | 0.628 | 1.03 (0.76, 1.41) | 0.838 |
| **Vital signs** | | | | |
| Temperature | 0.88 (0.77, 1) | 0.042 | 0.95 (0.84, 1.08) | 0.418 |
| Heart rate | 1.02 (1.01, 1.02) | <0.001 | 1.01 (1, 1.02) | 0.042 |
| Systolic BP | 1 (0.99, 1) | 0.201 | 1 (0.99, 1) | 0.531 |
| Systolic BP | 1 (0.99, 1.01) | 0.853 | 1.01 (1, 1.02) | 0.251 |
| SOFA | 1.19 (1.16, 1.23) | <0.001 | 1.2 (1.15, 1.25) | <0.001 |
| **Co-morbidities** | | | | |
| Hypertension | 0.77 (0.6, 1) | 0.047 | 0.74 (0.5, 1.09) | 0.13 |
| Myocardial infarction | 1.32 (0.95, 1.83) | 0.097 | 1.17 (0.82, 1.67) | 0.385 |
| Congestive heart failure | 1.62 (1.22, 2.15) | 0.001 | 1.13 (0.8, 1.59) | 0.491 |
| Diabetes | 0.89 (0.6, 1.32) | 0.572 | 0.83 (0.53, 1.29) | 0.4 |
| Renal failure | 1.71 (1.27, 2.31) | <0.001 | 0.99 (0.66, 1.49) | 0.972 |
| **Medications** | | | | |
| Statin | 0.43 (0.32, 0.58) | <0.001 | 0.76 (0.53, 1.07) | 0.119 |
| Anti-platelet drugs | 0.39 (0.3, 0.51) | <0.001 | 0.62 (0.45, 0.85) | 0.003 |
| ACEI/ARB | 0.31 (0.19, 0.51) | <0.001 | 0.56 (0.33, 0.97) | 0.038 |
| Beta-blockers | 0.30 (0.23, 0.39) | <0.001 | 0.52 (0.39, 0.7) | <0.001 |
| Vasopressor | 0.86 (0.67, 1.12) | 0.268 | 0.7 (0.49, 1.01) | 0.054 |
| Mechanical ventilation | 1 (0.73, 1.36) | 0.978 | 0.95 (0.59, 1.53) | 0.823 |
| GV | 5.33 (3.02, 9.4) | <0.001 | 2.19 (1.01, 4.74) | 0.047 |

**Abbreviations:** HR, hazard ratio; CI, confidence interval BP, blood pressure; SOFA, sequential organ failure assessment; ACEI/ARB, angiotensin converting enzyme inhibitors/angiotension receptor blockers; GV, glycemic variability.

**Note:** multivariable logistic analysis adjusted for: covariates included in demographics + vital signs + co-morbidities + medications.
